# Effects of a Combined Geothermal and Solar Heating System as a Renewable Energy Source in a Pig House and Estimation of Energy Consumption Using Artificial Intelligence-Based Prediction Model

**DOI:** 10.3390/ani12202860

**Published:** 2022-10-20

**Authors:** Hong-Seok Mun, Muhammad Ammar Dilawar, Shad Mahfuz, Keiven Mark B. Ampode, Veasna Chem, Young-Hwa Kim, Jong-Pil Moon, Chul-Ju Yang

**Affiliations:** 1 Animal Nutrition and Feed Science Laboratory, Department of Animal Science and Technology, Sunchon National University, Suncheon 57922, Korea; 2 Department of Multimedia Engineering, Sunchon National University, Suncheon 57922, Korea; 3 Department of Animal Nutrition, Sylhet Agricultural University, Sylhet 3100, Bangladesh; 4 Department of Animal Science, College of Agriculture, Sultan Kudarat State University, Tacurong City 9800, Philippines; 5 Interdisciplinary Program in IT-Bio Convergence System (BK21 Plus), Chonnam National University, Gwangju 61186, Korea; 6 Rural Development Administration, Jeonju 54875, Korea; 7 Interdisciplinary Program in IT-Bio Convergence System (BK21 Plus), Sunchon National University, 255 Jungangno, Suncheon 57922, Korea

**Keywords:** pig production, renewable energy source, energy efficiency, artificial intelligence, geothermal heat pump

## Abstract

**Simple Summary:**

A combined geothermal heat pump and solar system (GHPS) was installed at a pig house to check the effects on electricity consumption, greenhouse gas emission (GHE), internal farm temperature, the concentration of noxious gases and growth performance. The GHPS heating system reduced energy consumption and CO_2_ concentrations. Furthermore, the GHPS system effectively maintained the optimum temperature for pig growth inside the pigsty. Additionally, the artificial intelligence (AI)-based model ‘gene expression programming (GEP)’ was used to predict electricity consumption.

**Abstract:**

This experiment evaluated the performance of a combined geothermal heat pump and solar system (GHPS). A GHPS heating system was installed at a pig house and a comparative study was carried out between the environmentally friendly renewable energy source (GHPS) and the traditional heating method using fossil fuels. The impact of both heating systems on production performance, housing environment, noxious gas emission, and energy efficiency were evaluated along with the GHPS system performance parameters such as the coefficient of performance (COP), inlet and outlet water temperature and efficiency of solar collector. The average temperature inside the pig house was significantly higher (*p* < 0.05) in the GHPS heating system. Similarly, the outflow temperature was increased significantly (*p* < 0.05) than the inflow temperature. The results of COP and efficiency of the solar system also indicated that the GHPS is an efficient heating system. The electricity consumption and carbon dioxide gas concentration were also reduced (*p* < 0.05) in the GHPS system. This study also predicts electricity consumption using an artificial intelligence (AI)-based model. The results showed that the proposed model justifies all the acceptance criteria in terms of the correlation coefficient, root mean square value and mean absolute error. The results of our experiment show that the GHPS system can be installed at a pig house for sustainable swine production as a renewable energy source.

## 1. Introduction

Energy consumption and cost have been increasing all over the world because energy is important to social and economic development and better quality of life [1]. The excessive use of energy and its potential economic and environmental impacts in the livestock sector is gaining attention. As the world population and demand for food are increasing continuously, energy demands to sustain the dietary requirements for animal protein are also increasing [2]. During the winter season, it is critical to maintain the desired temperature inside the pig farm because they lack a thermoregulation process. Therefore, a recommendable growth environment inside the pig house should be provided using heating and ventilation systems [3]. It is reported by the Minnesota center that 5% of the pig production cost is needed for fuel and electrical consumption [4]. In addition, high energy usage contributes to environmental pollution and global warming [5]. A pig barn is a source of various air pollutants mainly including carbon dioxide (CO_2_), ammonia (NH_3_), particulate matter (PM_2.5_), endotoxins, and harmful microbes that contribute to global warming and environmental pollution [6]. The most prevalent greenhouse gas (GHG) is CO_2_, and the use of electricity results in the release of 32% of CO_2_ [1]. Similarly, the release of NH_3_ into the atmosphere is the primary source of atmospheric pollution linked to livestock and agriculture, consisting of 95% anthropogenic emissions [7]. Additionally, the emission of PM_2.5_ and total volatile organic compounds (TVOCs) is not only detrimental to the health of people and animals but also causes environmental pollution [8]. Therefore, it is a need of time to utilize renewable energy sources (RES) instead of fossil fuels in livestock farming for energy saving, sustainable livestock production, and environmental protection.

South Korea is one of the largest oil-consuming nations in the world. However, the usage of new and renewable energy (NRE) is very low (5% of overall energy use in 2011) as compared with many other countries such as the USA and Canada [9]. Therefore, the government in Korea is taking measures to increase the share of NRE to 11% by 2030. As a result, the Korean government is promoting the use of NRE by providing a 30–80% subsidy on installation costs. Renewable energy sources such as geothermal heat pumps (GHP), air heat pumps (AHP), and solar systems are gaining popularity in Korea. The Republic of Korea is considered to be blessed with huge reservoirs of geothermal energy resources (GER). It has been reported by Lee et al. [10] that the energy obtained from the GER in Korea would be equivalent to 200 times the predominant consumption of energy annually. The GER for livestock farms can be utilized at 100 to 500 m depth to drive GHP depending on the electricity load [6]. A GHP is considered to be an environmentally friendly, cost-effective, and efficient energy resource in livestock farms [11], as it can improve the air quality by providing fresh air and the GHP system does not produce combustion pollutants directly [12,13]. Previous studies have shown that by using the heating mode of the GHP system, 30–70% of energy consumption can be saved [11,12].

Solar energy is considered to be an ideal choice as NRE due to its high efficiency, abundant availability, inexhaustibility, and cost savings. Solar heating systems can be either photovoltaic driven or solar thermal [14]. By keeping in view the importance of solar energy (SE), the Korean Photovoltaic Industry Association (KOPIA) is trying to increase its applications in the agriculture sector including livestock farms [15]. Despite having many advantages, the supply of photovoltaic power or photo-thermal power is not continuous [16], so it could be harmful to pig farms because they need a continuous supply of energy for heating purposes [17]. Therefore, a supplementary energy supply including a thermal or electrical energy storage system should be installed to provide a continuous supply of energy to pig farms [18]. Several authors [11,12,14] have reported the experimental applications of individual (GHP and SE) renewable energy sources in pig farms for energy savings, growth performance, and housing environment. However, this experiment was performed to check the combined heating effects of GHPS on pig growth performance, noxious gas concentrations, and energy consumption in a pig house. Additionally, an artificial intelligence (AI)-based prediction model to estimate electricity consumption at pig farms was developed using a gene-expressing programming (GEP) machine learning approach.

## 2. Materials and Methods

### 2.1. Housing and Animal Care

**Figure 1:**
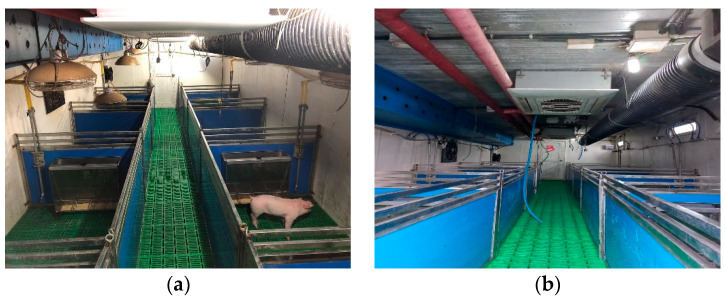
Inside pictures of pig houses used in this study. (**a**) Control house with heating lamps of adjustable height. (**b**) Pig house installed with GHPS heating system.

This trial was carried out at the research farm of Sunchon National University, Suncheon, the Republic of Korea from 18 March 2022 to 5 May 2022 for 7 weeks. A total of 20 pigs [(Landrace × Yorkshire) × Duroc] having an average initial body weight of 13 kg were reared in two separate houses (8.2 m length × 3 m width), which were further separated into ten individual replications. One pig house was equipped with a traditional heating system (10 heating lamps of 600 W each having an adjustable height according to the age of pigs were placed on each pen’s top) and was considered a control (Figure 1a). The other pig house was installed with a combination of a geothermal heat pump and solar photovoltaics (GHPS) (Figure 1b).

All animals were kept and reared on a slatted floor and provided a commercial diet and fresh water. The feed composition of the diet is the same as reported in our previous study [11].

### 2.2. Growth Performance

The animals were weighed individually every week and weight gain was calculated. Similarly, the feed intake (FI) was recorded by collecting the refused feed from the feed provided. The feed conversion ratio (FCR) was measured by dividing the feed eaten, by the live weight gained.

### 2.3. Consumption of Electricity and CO_2_ Concentration

The electricity consumption for heating the pig house was calculated by two separate smart energy electric sub-meters. The concentration of CO_2_ was checked by installing the Bandiburri smart farm monitoring system (NareTrend, Inc., Bucheon City, Korea).

### 2.4. Inside and Outside Temperature Measurement and Outflow and Inflow Temperature

The temperature inside both pig houses and the outside temperature were recorded by SMT-75, T-type thermistors, and thermocouples (Seoul semiconductors, Seoul, Korea) having a range of −20 °C to 80 °C. The outflow and inflow (hot and cold water) temperature of the solar collector tubes and GHP system was measured by a GPT-1000 pipe temperature sensor (Ginice Co. Ltd., Bucheon, Republic of Korea), having a high-quality thermistor sensor element having a range from −50 °C to 150 °C.

### 2.5. Hydrogen Sulfide (H_2_S) and Ammonia (NH_3_) Concentration

The concentration of both noxious gases (H_2_S and NH_3_) was checked by fitting the sensor to the ceiling (at a height of 1.8 m) of the pig house. The sensor used for ammonia was NH_3_-3E 100 SE (City Technology, Bonn, Germany) and the sensor used for hydrogen sulphide was H_2_S-B4 (Alphasense Ltd., Great Notley, UK). To verify the reading from sensors, the concentration of NH_3_ and H_2_S was also measured weekly by using a GV-100 gas sampling pump and detection tubes 3 L for NH_3_ and 4 LT for H_2_S (Gastec Corp, Ayase-Shi, Japan).

### 2.6. Formaldehyde, Total Volatile Organic Compounds (TVOCs) and Particulate Matter (PM_2.5_)

The concentrations of formaldehyde (FA), PM_2.5_, and TVOCs were measured by using an air quality smart sensor, AR830A (SmartSensor, Dongguan, China). The sensor ranges for FA, PM_2.5_, and TVOC are 0–5 ppm, 0–5 ppm, and 0–150 µg/m^3^, respectively.

### 2.7. Description of the GHPS System

**Figure 2:**
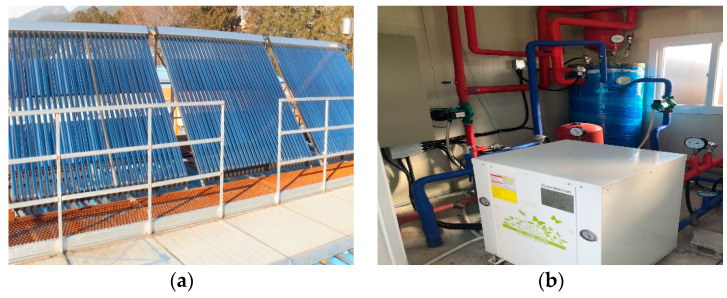
Heating systems used in the experiment. (**a**) Evacuated tube collector. (**b**) Geothermal heat pump system with circulating pipes and water storage tank.

The solar system Apricus AP-30 solar (Apricus Solar Ltd. Jiangsu, China) comprised of 30 evacuated tube collectors was installed on the roof of a pig house in one array at an angle of 60 °C in the south-facing direction to collect heat energy from solar radiations (Figure 2a). The collector panel covered a total area of 15.75 m^2^. The glass tubes (58 mm diameter each) have a copper pipe (served as heat pipe) that can transfer solar heat through the convection process of heat exchange fluid present inside the hot bulb and indirectly gives heat to a copper manifold (heat exchanger). All copper pipes were combined to a common manifold attached to a storage tank of 300 L capacity (having water for heating). A lubricant oil (propylene glycol) was used as a heat transfer liquid to avoid freezing at reduced temperatures and a regulatory pump was used to control oil circulation. The hot water was circulated from the storage tank using a regulatory pump via copper pipes (having 9.52 mm diameter) attached to the longitudinal wall of the experimental pig farm. This allows the transfer of heat to the pig farm by radiation process. After that, the cold water was transferred to the water tank that cooled down the oil in copper pipes of 19.05 mm in diameter. Finally, the cold oil was again transferred to the evacuated tube collector to gather heat through solar energy.

**Figure 3:**
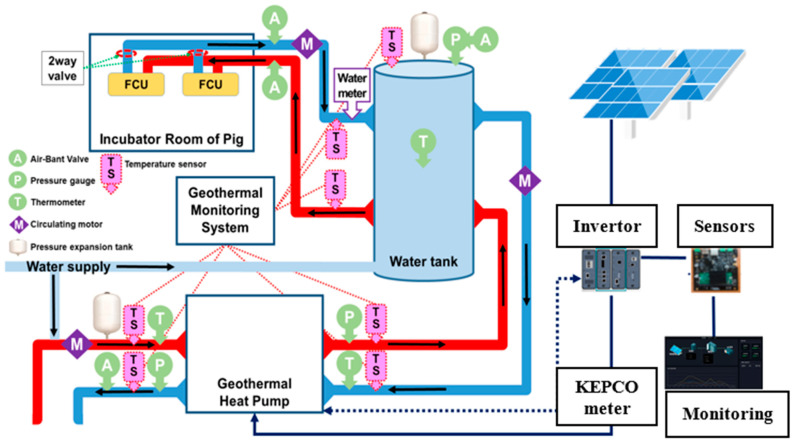
Combined geothermal and solar heating system flow diagram.

A ground source DHGW 5NC402 geothermal heat pump (Daesung Heat Enersys, Seoul, Korea) with a heating and cooling capacity of 19.66 kW and 20.59 kW, respectively, having a 4.93 kW rating electrical power consumption (when operated under optimum conditions) was installed and connected to the experimental pig house (Figure 2b). The GHP comprised a borehole exchanger (BHE, 150 m deep double U tube), fan coil unit (FCU), water circulating pumps, and a thermal tank. A total of three circulating pumps were attached to the system to transfer water from the ground to the heat pump unit (Model PH200M, Wilo Pump, Ansan, Korea with a 135 L/min flow rate) from the heat pump to the water storage tank (Model PH080M with a 75 L/min flow rate) and from the water tank to the inside of pig farm (Model PB600MA with an 80 L/min flow rate). A thermal water tank of 260 L storage capacity was used to keep high-temperature water. The heat was transferred to the pig farm from the water tank through a fan coil unit (FCU). In the heat pump, an environmentally safe working fluid R-407A was used. A schematic flow sheet diagram of the combined geothermal heat pump and solar heating system is shown in Figure 3.

### 2.8. Solar System Efficiency and Geothermal Heat Pump Coefficient of Performance

The efficiency of the solar collector was determined by using Equation (1) explained by Islam et al. [19].

(1) $${ƞ}_{\mathrm{collector}}={ƞ}_{o}-{\;\alpha }_{1}\left[\frac{\left(T_{m}-T_{a}\right)}{G}\right]-{\;\alpha }_{2}\left[\frac{{\left(T_{m}-T_{a}\right)}^{2}}{G}\right],\;$$

where ƞ_collector_ = solar collector efficiency; ƞ_o_ = optical efficiency; α_1_ = coefficient of first order heat loss; α_2_ = coefficient of second order heat loss; T_m_ = collector temperature; T_a_ = air ambient temperature, and G = solar radiation (W/m^2^).

The output of the solar collector and the coefficient of performance of GHP was calculated by using Equations (2) and (3).

Energy output (kWh/m^2^/d) = Solar radiation (kWh/m^2^/d) × Collector efficiency × Aperture area (m^2^) (2)

(3) $$\mathrm{COP}=\frac{\mathrm{Power}\;\mathrm{consumption}\;\left(\mathrm{kW}\right)+\mathrm{Absorbed}\;\mathrm{heat}\;\left(\mathrm{kW}\right)}{\mathrm{Power}\;\mathrm{consumption}\;\left(\mathrm{kW}\right)},$$

where absorbed heat = M × C_p_ × ΔT × 4 ÷ 3600 and M = mass flow rate (kg/h); C_p_ = specific heat in kcal/kg °C; and ΔT = difference between outlet and inlet temperature.

### 2.9. Development of the Prediction Model and Pre-Processing of Data

The prediction model for the estimation of electricity consumption (Ec) was developed using GEP. The prediction model was further validated and verified by different statistical check and error graph plots with parametric and sensitivity studies. In this experiment, the most critical parameters were included for the development of the prediction model. The parameters include temperature (T), humidity (H), ventilation rate (V), ammonia concentration (Ac), CO_2_ concentration (Cc), heating load by pig’s weight (Hl), and concentration of PM_2.5_ (Pc). Therefore, the Ec is considered to be the function of several parameters of the pig house as given in Equation (4).

(4) Ec=f(T,H,V,Ac,Cc,Hl,Pc)

The dataset from 196 samples was divided into two groups; i.e., for training purposes (142 samples) and validation testing (54 samples).

#### 2.9.1. Gene Expression Programming Model

The GEP model was first proposed by Ferreira [20] and is widely used in geotechnical engineering-related fields. It has several advantages over other similar techniques such as artificial neural networking (ANN) because of its transparent mathematical solutions to problems [21]. The prediction model in GEP depends on many factors including head size, operators, linking function, and number of expression trees (ET) or genes (output equations). The details of general settings for different parameters used in the development of the prediction model for our experiment are shown in Table 1.

As shown in Table 1, three expression trees A, B, and C (Equations (6)–(8)) were calculated to derive Equation (5) for the prediction and evaluation of Ec using the Karwa language explained by Koza and Poli [22].

E_c_ = A + B + C, (5)

(6) $$A=\mathrm{do}-\frac{d3+11.57}{10.95-d5+d2}+\mathrm{do}+3.27$$

(7) $$B=(\left(\frac{-8.66}{\mathrm{do}\times \left(-8.66\right)}-\mathrm{do}-d3\right)\times \left(-3.59-2.0-d1\right))\times \left(-7.17\right)$$

(8) $$C=\frac{d3}{2.07}(\left(\frac{\frac{d3}{2.07}}{d0}\right)-\left(2.07+3.11\right)-\left(2.07-d2\right)-\left(d4\times d6\right)-3.11),$$

where do = temperature (T), d1 = humidity (H), d2 = ventilation rate (V), d3 = ammonia concentration (Ac), d4 = CO_2_ concentration (Cc), d5 = heating load by pig’s weight (Hl), and d6 = concentration of PM_2.5_ (Pc).

#### 2.9.2. Performance Evaluation of Prediction Model

In this experiment, statistical tests such as mean absolute error (MAE), root mean square value (RMSE), and correlation coefficient (R or R^2^) were used for the validation of the model as shown in Equations (9)–(11), respectively.

(9) $$R=\frac{\sum_{i=1}^{n}(G_{\mathrm{si}}^{\exp}-\bar{G_{\mathrm{si}}^{\exp}})\left(G_{\mathrm{si}}^{\mathrm{pred}}-\bar{G_{\mathrm{si}}^{\mathrm{pre}}}\right)}{\sqrt{\sum_{i=1}^{n}{\left(G_{\mathrm{si}}^{\exp}-\bar{G_{\mathrm{si}}^{\exp}}\right)}^{2}}\times \sum_{i=1}^{n}{\left(G_{\mathrm{si}}^{\mathrm{pred}}-\bar{G_{\mathrm{si}}^{\mathrm{pred}}}\right)}^{2}\;}$$

(10) $$\mathrm{RMSE}=\sqrt{\frac{\sum_{i=1}^{n}{\left(G_{\mathrm{si}}^{\exp}-G_{\mathrm{si}}^{\mathrm{pred}}\right)}^{2}}{n}}$$

(11) $$\mathrm{MAE}=\overset{n}{\underset{i=1}{\sum }}\frac{\left|G_{\mathrm{si}}^{\exp}-G_{\mathrm{si}}^{\mathrm{pred}}\right|}{n}\;,$$

where n = no of samples; $G_{\mathrm{si}}^{\exp}$ = ith value of modulus from experimental data; $G_{s_{i}}^{\mathrm{pred}}$= ith predicted model output, and $\bar{{G_{s}}_{i}^{\exp}}$ and $\bar{{G_{s}}_{i}^{\mathrm{pred}}}$ = mean values of experimental and the model outputs of Ec respectively.

### 2.10. Statistical Analysis

All experimental data were also tested using the Statistical Package for Social Science (SPSS program, version 15.1, Chicago, IL, USA). All parameters were analyzed between treatments by one-way ANOVA and subsequent Tukey’s post hoc test. The following Equation (12) was used to test the effects:

Yij = µ + α_i_ + e_ij_, (12)

where Y_ij_ represents the response variable, µ is the mean value, α_i_ shows the effect of dietary treatments and e_ij_ is the error. The probability values lower than 0.05 were considered significant.

## 3. Results

### 3.1. Growth Performance

The effects of the heating system on pig growth performance are presented in Table 2. There was no significant difference (*p* > 0.05) observed in the weight gain and feed intake between the energy systems. However, the weight gain in the pig house installed with GHPS increased non-significantly as compared to the control. Similarly, the FCR in both groups showed no significant differences.

### 3.2. Electricity Consumption and CO_2_ Concentration

The electricity consumption was reduced significantly (*p* < 0.05) in the pig house heated by the GHPS as compared to the traditional heating system (Table 3). The energy savings were 31.58% in the GHPS system. Similarly, the CO_2_ concentration was reduced significantly in the GHPS-installed pig house relative to the control (traditional heating system).

### 3.3. Pig House Inside Temperature and Outflow and Inflow Temperature

**Figure 4:**
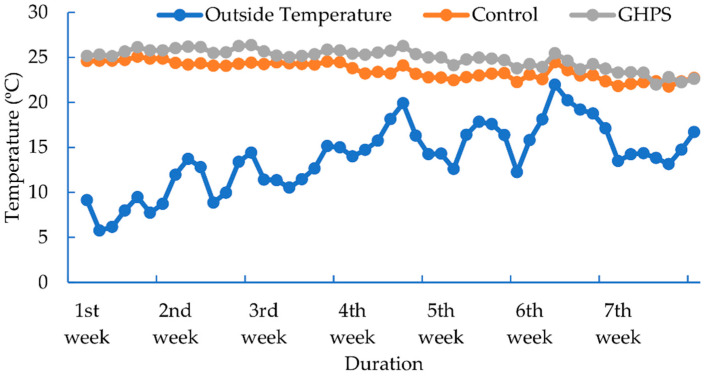
The temperature of the outside, control, and combined geothermal and solar heating system (GHPS).

The overall temperature pattern during the experiment for the control and GHSP is presented in Figure 4. The temperature was adjusted at 26 °C during the first week then decreased at the rate of 1 °C weekly and maintained at 20 °C afterwards. The average temperature in the GHPS pig house was significantly higher than the outside and traditional heating system. The mean temperature was increased by 77.53% in the GHPS-connected pig house relative to the outside and by 5.9% compared to the control temperature.

**Figure 5:**
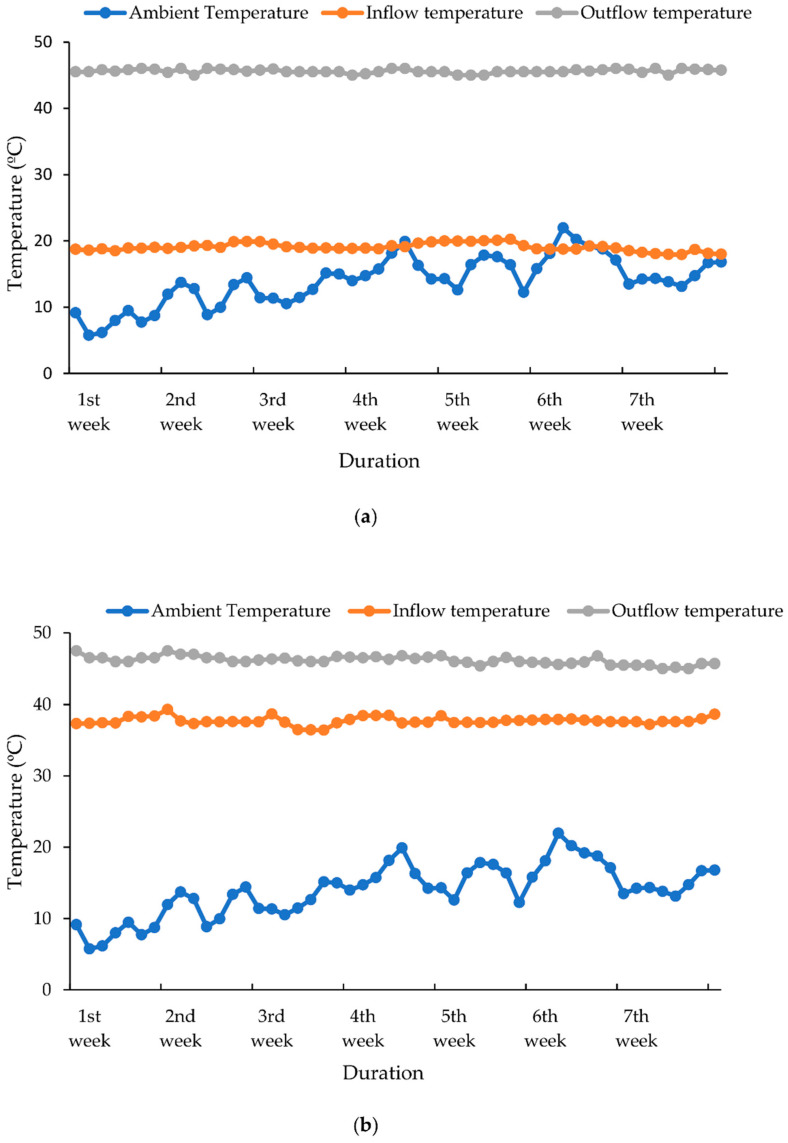
Outflow and Inflow temperature (**a**) GHP outflow and inflow temperature. (**b**) Solar collector outflow and inflow temperature.

The outflow temperature for the GHP system (Figure 5a) and solar collector (Figure 5b) was significantly (*p* < 0.05) greater than the inlet temperature throughout the experiment. The mean temperature difference between the outflow and inflow temperature was 9.

### 3.4. Concentration of NH_3_, H_2_S, FA, PM_2.5_ and TVOC

There was no difference statistically (*p* > 0.05) in the concentration of ammonia in both houses heated with the GHPS system and control heating system. The concentration of H_2_S was not detectable in both houses (data not shown). Similarly, the concentration of TVOC and FA did not show any significant differences (Table 4). However, the concentration of PM_2.5_ was reduced (*p* < 0.05) in the pig house installed with GHPS relative to the control.

### 3.5. COP of GHP and Solar System Collector’s EfficiencyTVOC

The energy output, solar intensity, and collector efficiency of the solar system and COP of the GHP system are presented in Table 5. The average highest solar intensity and collector efficiency was recorded in the third week, while the minimum value was found in the first week. Similarly, the COP of GHP was higher in the third week of the observed period.

### 3.6. Performance Evaluation of the GEP Model

**Figure 6:**
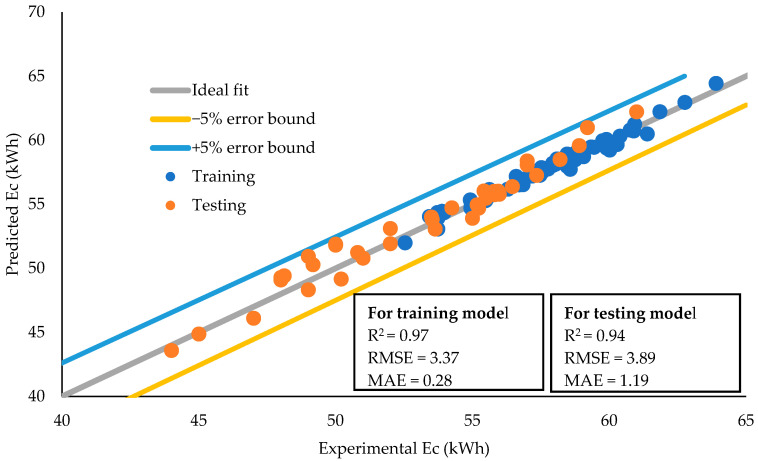
Performance assessment of prediction models. The statistics of the regression line of GEP model against training and testing data.

The results of the GEP model for the estimation of Ec of the heat pump are shown in Figure 6. The value of statistical test parameters such as RMSE, MAE, and R^2^ was 3.37, 0.28, and 0.97 for the training model and 3.89, 1.19, and 0.94 for the validation model, respectively.

**Figure 7:**
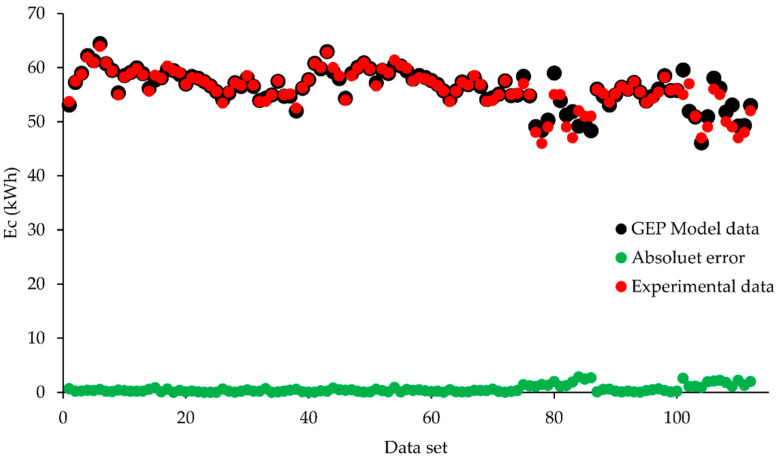
Absolute error plot for GEP prediction model to compare absolute error.

The error plots between observed and predicted values are presented in Figure 7. It indicates the absolute error of the experimental data utilized for training the GEP prediction model and response. The results show that the average value of absolute error is 1.81 kWh which is minimal.

**Figure 8:**
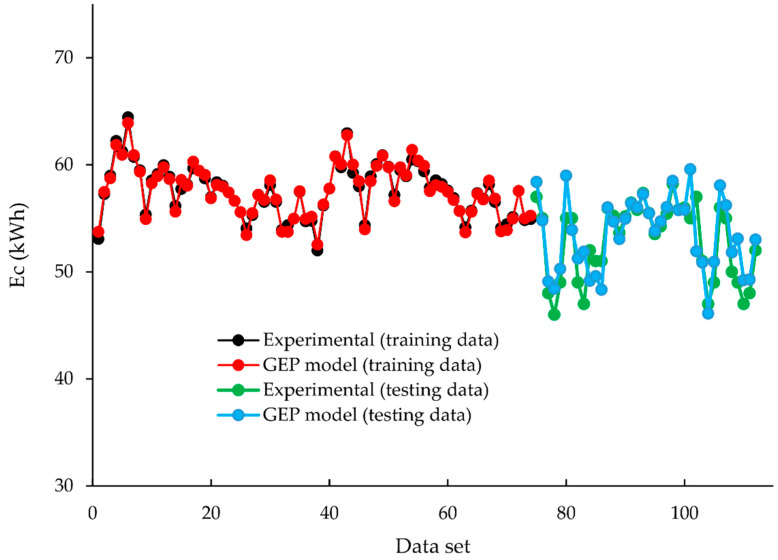
Comparison of the experimental and predictive dataset for training and testing phase based on GEP model.

The predicted and experimental responses of the GEP model are shown in Figure 8. It can be observed that the predicted and experimental responses overlap with each other representing the strong coherency and correlation between the experimental and predicted response.

## 4. Discussion

The energy shortage problem at the global level is threatening most economies around the world becoming more critical with time. The implementation of renewable energy resources (RER) in livestock production can reduce the reliance of global food production (animal protein source) on fossil fuels [11]. The most important parameters in pig production for farmers are market weight and feed efficiency [23] and these parameters must be given top priority before implementing any new technology in pig farming. In our experiment, the GHPS system had no harmful effects on the pig performance relative to the traditional heating system methods. Similar results were reported by previous studies [6,11,14,19] that GHP and solar system (either alone or in combination) did not affect the growth performance and feed intake in pigs.

The efficiency of a solar system is dependent on the solar intensity per square foot (ft^2^) of a specific area or region [24]. Several factors including seasonal changes, temperature, relative humidity, and weather conditions of a particular area can affect the performance of solar collectors [25]. Geothermal energy is one of the world’s most important renewable energy sources for power generation and the source of heat (underground water) for a heat pump system is determined by local availability, ambient temperature, water temperature, level of groundwater, thermal conductivity, and climatic circumstances [26,27]. In our experiment, there were fluctuations in the number of solar radiations every week. The high radiations were observed on sunny bright days having low humidity levels while low radiations were noticed on cloudy or humid days. These values are in agreement with the results reported by Islam et al. [19] that the intensity of solar radiation reduces with high humidity levels due to the high attenuation by water molecules. Similarly, the heating capacity of the GHP exchanger is also affected by temperature fluctuations but in the surface zone [28].

One of the important performance and efficiency evaluators of the GHP system is the coefficient of performance (COP). The optimum value of COP ranges from 4.19 to 4.57 for the GHP heating mode in winter [29]. The calculated values of COP in our study for GHP are in the normal range and are close to the values calculated in the previous experiments by Tong et al. [30] and Sanner et al. [31]. Similarly, the better efficiency of the solar system’s collector was found in this experiment. This could be attributed to higher solar radiation at that particular time of the study. The increased sunlight intensity may boost the efficiency of the collector and output of energy by decreasing the temperature difference between the ambient temperature and the collector’s fluid [32]. Owning to the better COP of GHP and high efficiency of solar system’s collector, they are gaining popularity as a renewable alternative source to traditional heating methods [14,33].

Environmental parameters have a significant role in pig productivity, and fluctuations in these conditions reduce animal performance drastically. The temperature inside the pig farm is one of the challenging housing parameters, that is crucial to the pig growth and production performance [34]. The atmospheric temperature and difference in temperature between the inlet and outlet fluid are the primary indicators to determine the efficiency and functionality of heating systems to maintain the desired temperature inside the buildings [19]. In our experiment, there was a significant difference in the fluid temperature of the inlet and outlet for the geothermal heat pump and solar system. The increased temperature of water at outflow indicates the efficiency of both systems to provide enough heat to keep the optimum temperature of the pig house during the study. These results are in agreement with previous studies that the high difference between ambient temperature and solar collectors (in the case of the solar system) and the significant difference between outlet and inlet water temperature of the heat pump (in the case of GHP) is crucial for the efficiency of heating systems [11,14]. Similarly, the inside temperature of the pig house was higher in the GHPS system due to the difference in the inlet and outlet temperature relative to the traditional system. Geothermal and solar heating systems are capable of continuous heat supply and uniform distribution. It was reported that the single loop vertical GHP system (known as the direct heat exchange system) can efficiently transfer heat from the ground to the source building and the fan coil unit (FCU) of the GHP system can distribute that heat effectively due to the continuous motion [19,33]. In line with our findings, many scientists from different nations have explained the satisfactory heating capabilities of the GHP system for animal farms [6,19,35,36].

During the winter season, farmers typically utilize electricity from fossil fuels burning, natural gas, or furnace oil for heating animal farms, which can emit fumes and increase noxious gas emissions. The emission of noxious gases from feces is also a major concern in animal farms because they contribute to pollution and livestock health issues [19]. The release of NH_3_ is also affected by the inside environment of livestock facilities, and concentration is positively connected with ambient temperature and ventilation rates [37]. Similarly, PM_2.5_ and FA concentrations in the pig farm have a significant impact on the respiratory system of animals and humans, as well as the environment [38]. In our experiment, no significant reduction was observed in the concentration of formaldehyde and TVOC. The concentration of NH_3_ was reduced (although non-significant) in the GHPS system. Similarly, the concentration of PM_2.5_ was according to the normal range of Korean ambient air quality in the GHPS-installed pig house. The GHPS system is considered to be efficient in reducing noxious gas emissions because it produces no fumes such as burning fossil fuels. Additionally, the reduced heating hours and continuous exchange of inside air with fresh air (by sickle FCU) can decrease the concentration of harmful gases inside swine farms [39].

The expenses associated with heating livestock facilities are challenging for pig producers as the price of fossil fuels is increasing continuously throughout the world. Renewable energy sources are gaining popularity because they are considered to be energy-efficient, environmentally friendly, abundantly available, and less costly [40,41]. In our experiment, the electricity usage was significantly reduced in the GHPS system as compared to the traditional heating system. The reason behind the low electricity consumption is because of the reason that GHP and the solar system use RER, while the traditional system uses fossil fuel combustion to generate electricity. It was also reported by Charoenvisal [42] that the GHP system consumes a single unit of electricity to produce 3 units of geothermal energy. The energy efficiency and cost-saving effects of solar-assisted GHP systems have been well-reported in many studies [14,19]. Similarly, the substantial reduction in electricity usage was linked with a significant decrease in CO_2_ emission in the GHPS system, which was also observed in our study. Nakomcic-Smaragdakis and Dragutinovic [43] have explained the three reasons which contribute to the reduction in CO_2_: carbon dioxide emission factor for the source of electricity, efficiency of technology, and less operating hours, and coefficient of performance (COP) of heat pumps. Electricity consumption by burning fossil fuels is the second largest contributor (26.9%) to GHE, which can cause health and environmental issues [44]. Therefore, environmental protection agencies are focusing on the use of alternative energy sources to reduce GHE. It can be concluded that the GHPS system can reduce the electricity cost and GHE with a 50% reduced operational and maintenance expenses [11]. However, due to the high installation costs of these systems, the subsidies from governments for farmers are necessary to promote the use of RER for saving the energy and environment [45].

## 5. Conclusions

In conclusion, the GHPS heating system decreased the consumption of electricity and CO_2_ concentration in the pig house. The GHPS system also efficiently maintained the desired inside temperature required for optimum pig production. Therefore, it is recommended that food production should be combined with renewable energy systems to decrease the dependency on fossil fuels and enhance food security and save the environment. However, the financial support of governments for installing the renewable energy system is necessary for the farmers due to the high installation costs.

## Figures and Tables

**Table 1:** General settings for prediction model.

Items	Model Setting
	Title 2
Genes (expression trees)	3
Chromosomes	200
Head size	10
Set of functions	+, −, ×, ÷
Linking functions	+

**Table 2:** Effect of a combined geothermal and solar heating system (GHPS) on the growth parameters.

Parameters	Control	GHPS	SEM	*p*-Value
Initial body weight (kg)	13.00	12.98	0.671	0.981
Final body weight (kg)	40.73	42.00	1.161	0.758
Weight gain (kg)	27.73	29.01	0.660	0.281
Feed intake (kg)	55.77	58.44	1.075	0.692
FCR	1.38	1.42	0.027	0.854

**Table 3:** Effect of the combined geothermal and solar heating system (GHPS) on electricity consumption (kWh) and CO_2_ concentration (kg).

Periods	Electricity Use		Reduced	CO_2_ Emission		Reduced
	Control	GHPS		Control	GHPS	
0–4 weeks	2055 ^a^	1622 ^b^	433	1179 ^a^	741 ^b^	438
4–7 weeks	1384 ^a^	1091 ^b^	293	757 ^a^	499 ^b^	258
Total	3439 ^a^	2713 ^b^	726	1936 ^a^	1240 ^b^	1501

^a, b^ Values with different alphabets differ significantly.

**Table 4:** Effect of the combined geothermal and solar heating system (GHPS) on formaldehyde (ppm), particulate matter (µg/m^3^), total volatile organic compounds (µg/m^3^), and ammonia concentration (ppm).

Items	Control	GHPS	SEM	*p*-Value
Formaldehyde	0.04	0.03	0.031	0.053
Particulate matter	36.10 ^a^	34.01 ^b^	0.017	0.034
Total volatile organic compounds	125	125	0.036	0.814
Ammonia	2.58	2.49	0.029	0.981

^a, b^ Values with different alphabets differ significantly.

**Table 5:** Coefficient of performance (COP) of the GHP system and calculated collector’s efficiency and energy output of evacuated tube collectors of the solar system.

	Ambient Temperature (°C)	Solar Intensity (W/m^2^)	Efficiency of Collector (%)	Output of Energy (kWh/m^2^/d)	COP of GHP
1st week	7.85	1154.88	61.45	210.62	4.35
2nd week	12.15	1191.35	60.40	172.29	4.60
3rd week	12.51	1582.83	65.01	208.19	4.98
4th week	16.15	1396.27	63.08	188.14	4.35
5th week	15.33	1262.32	62.95	173.01	4.47
6th week	18.73	1451.50	64.90	198.31	4.83
7th week	14.34	1362.23	64.10	187.90	4.73

## Data Availability

Not applicable.

## Nomenclature

AHP	Air heat pump
AI	Artificial intelligence
ANN	Artificial neural networking
ANOVA	Analysis of variance
AQS	Ambient air quality standards
BHE	Bore hole exchanger
BWG	Body weight gain
Cc	CO_2_ concentration
COP	Coefficient of performance
CO_2_	Carbon dioxide
C_p_	Specific heat (kcal/kg °C)
Ec	Electricity consumption
ET	Expression trees
FA	Formaldehyde
FCR	Feed conversion ratio
FCU	Fan coil unit
FI	Feed intake
G	Solar radiation
GEP	Gene expression programming
GER	Geothermal energy resources
GHG	Greenhouse gas
GHP	Geothermal heat pump
GHPS	Combined geothermal heat pump and solar system
H	Humidity
HI	Heating load
H_2_S	Hydrogen sulphide
KOPIA	Korean Photovoltaic Industry Association
kW	Kilowatt
kWh	Kilowatt hour
M	Mass flow rate (kg/h)
MAE	Mean absolute error
NH_3_	Ammonia
NRE	New renewable energy
Pc	Concentration of PM_2.5_
PM_2.5_	Particulate matter
R	Correlation coefficient
RER	Renewable energy resource
RMSE	Root mean square value
SE	Solar energy
SPSS	Statistical package for social science
T	Temperature
T_m_	Mean collector temperature
T_a_	Ambient temperature
TVOC	Total volatile organic compounds
ΔT	Inlet˗outlet temperature difference (°C)
V	Ventilation rate
α_1_	Coefficient of first-order heat loss
α_2_	Coefficient of second-order heat loss
µ	General mean
α_ij_	Effect of treatment
e_ij_	Random error
Y_ij_	Response variable
ƞ_collector_	Solar collector efficiency
ƞ_o_	Optical efficiency
